# Dietary practices and nutritional status of adult pulmonary tuberculosis patients in Kiambu level five hospital, Kiambu county, Kenya

**DOI:** 10.1186/s40795-026-01346-9

**Published:** 2026-07-08

**Authors:** Isaac Nthiga, Judith Kimiywe, Dorcus Mbithe David-Kigaru

**Affiliations:** 1 Department of Human Nutrition, Kabarak University, Nakuru, Kenya; 2 Department of Food, Nutrition and Dietetics, Kenyatta University, Nairobi, Kenya; 3 Department of Human Nutrition, P.O Box 2868-00100, Nairobi, Kenya

**Keywords:** Tuberculosis, Nutritional Status, Dietary Practices, Meal Frequency, Nutrient Adequacy, Body Mass Index, Food Consumption Patterns

## Abstract

**Background:**

Tuberculosis (TB) remains the second leading cause of death from an infectious disease after COVID-19, with Kenya among the high-burden countries. Kenya ranks 14th globally and 5th in Africa in TB burden. TB significantly affects dietary practices and nutritional status, which in turn influence treatment outcomes. However, limited data exist on dietary practices and nutritional status of TB patients in specific settings such as Kiambu County. This study aimed to assess the dietary practices and nutritional status of adult pulmonary TB patients at Kiambu Level Five Hospital, Kiambu County, Kenya.

**Methods:**

An analytical cross-sectional study was conducted among 141 TB patients selected through systematic sampling at the Kiambu Level Five Hospital TB clinic. Data on dietary practices, including meal frequency, food consumption patterns, and nutrient adequacy, were collected using a researcher-administered questionnaire and a 24-hour dietary recall. Body Mass Index (BMI) was used to assess nutritional status. Chi-square tests were used to determine associations between categorical variables, while Pearson’s product–moment correlation coefficient assessed relationships between continuous variables. Multiple linear regression analysis was performed to identify independent predictors of nutritional status, adjusting for relevant socio-demographic and dietary factors.

**Results:**

The mean age of participants was 33.9 ± 12.6 years, with the majority being male (59.6%) and aged 25–34 years. Multiple linear regression analysis identified meal frequency (β = 1.22, *p* < 0.001) and total energy intake (β = 0.003 per kcal, *p* < 0.001) as significant positive predictors of BMI. Regular lunch consumption and older age were also associated with higher BMI, while living with others was associated with lower BMI. The final model explained 42% of the variance in nutritional status (Adjusted R² = 0.42).

**Conclusion:**

Dietary practices, particularly meal frequency and energy intake, are key determinants of nutritional status among TB patients. Interventions focusing on improving dietary intake and addressing socio-demographic factors may enhance nutritional recovery and treatment outcomes in this population.

**Supplementary Information:**

The online version contains supplementary material available at 10.1186/s40795-026-01346-9.

## Background

Tuberculosis (TB) remains a major global health challenge, ranking as the second leading cause of death from an infectious disease after COVID-19 [1]. Despite the availability of effective treatment, TB continues to be a significant public health burden, particularly in low-and middle-income countries. Kenya is among the 30 high-TB-burden countries globally, with approximately 140,000 new TB cases reported annually (Ministry of Health, [2]). Within Kenya, the burden of tuberculosis varies across counties. Nairobi County reports the highest proportion of TB cases nationally, accounting for approximately 13% of notified TB cases, while Kiambu County contributes about 5%, according to reports by the Ministry of Health (Ministry of Health, [3]).

The persistence of TB in these regions underscores the need for a comprehensive approach to management, including both medical treatment and nutritional interventions.

Nutrition plays a critical role in TB prevention, management, and recovery. Malnutrition weakens the immune system, increasing susceptibility to TB infection, while TB itself contributes to undernutrition by elevating metabolic demands, suppressing appetite, and impairing nutrient absorption [4]. Studies show that undernourished TB patients experience more severe disease progression, higher treatment failure rates, and increased mortality compared to those with adequate nutrition [1]. Proper dietary intake, particularly a balanced diet rich in proteins, vitamins, and minerals, strengthens immunity and improves treatment outcomes (World Health Organization, [5]; [6]).

Despite the well-established link between nutrition and TB, dietary practices among TB patients remain suboptimal, with many experiencing food insecurity, irregular meal patterns, and insufficient nutrient intake. In Kenya, studies have reported that TB patients frequently skip meals due to financial constraints, lack of appetite, or side effects of medication [7]. Reliance on starchy staple foods with limited protein and micronutrient intake exacerbates malnutrition. Limited data exist on the specific dietary practices and nutritional status of TB patients in Kiambu County, hindering targeted interventions.

This study aimed to assess the dietary practices and nutritional status of adult pulmonary TB patients at Kiambu Level Five Hospital, examining the association between meal frequency, meal pattern, food consumption pattern and nutrient adequacy with nutritional status. Findings may inform nutritional support strategies for TB patients.

## Methods

### Study design and setting

This study adopted an analytical cross-sectional design to assess dietary practices and nutritional status among adult pulmonary tuberculosis (TB) patients. Cross-sectional study designs are commonly used to examine relationships between exposures and health outcomes at a specific point in time. In this study, an exploratory analytical approach was applied to examine potential associations between dietary practices including meal frequency, food consumption patterns, dietary diversity, and nutrient intake and the nutritional status of adult pulmonary TB patients.

The study was conducted at Kiambu Level Five Hospital (KLFH) in Kiambu County, Kenya. The facility was selected due to its relatively high TB burden and substantial patient volume, making it suitable for recruiting adult pulmonary TB patients. Data collection was carried out between May and June 2023.

### Study population

The study targeted adult pulmonary TB patients aged 18 years and above who were receiving treatment at KLFH. Patients were included in the study if they were diagnosed with pulmonary TB, aged 18 years and above, and willing to provide informed consent. Patients were excluded if they had extrapulmonary TB or were too ill to participate in the study.

### Sampling

A systematic random sampling method was used to select 141 participants from the hospital’s TB clinic records. The sampling interval of 3 was determined by dividing the estimated number of TB patients attending the clinic within the study period by the required sample size. Every 3rd patient from the register was selected until the sample size was achieved.

### Sample size determination

The sample size was determined using G*Power software version 3.1.9.7, based on an 80% power, a 0.05 alpha level, and a moderate effect size of 0.25. The computed sample size was 120, with a 20% adjustment for potential dropouts, resulting in a target sample of 144.

### Data collection tools and procedures

#### Questionnaire and dietary assessment

A structured researcher-administered questionnaire was used to collect data on demographic and socio-economic characteristics, meal frequency, food consumption patterns, and nutrient adequacy. A 24-hour dietary recall was used to assess nutrient intake, capturing all food and beverages consumed the previous day. To minimize recall bias, participants were prompted using food models, portion-size visuals, and probing questions for accuracy.

#### Nutritional assessment

Body Mass Index (BMI) was used to assess nutritional status, calculated as weight (kg) divided by height (m²). The WHO BMI classification was used: BMI < 18.5 – Underweight, BMI 18.5–24.9 – Normal weight and BMI ≥ 25 – Overweight.

### Recruitment and training of research assistants

Two research assistants with backgrounds in nutrition and dietetics were recruited and trained to ensure consistent data collection. The training covered study objectives, ethical considerations, interviewing techniques, and anthropometric measurements. Hands-on practice with mock interviews and demonstrations was conducted before fieldwork began to ensure competency.

### Pre-testing and instrument validation

The questionnaire was pre-tested among 15 TB patients at Thika Level Five Hospital (10% of the study sample). This process helped refine unclear questions before full implementation. Additionally, three nutrition experts reviewed the questionnaire, and their feedback was incorporated to enhance content validity.

### Reliability testing

A test-retest method was used to assess questionnaire reliability. The same questionnaire was administered twice, seven days apart, to the same 15 participants at Thika Level Five Hospital. Responses were compared using Pearson’s correlation coefficient, with a value above 0.8 considered acceptable for reliability. For anthropometric reliability, weight and height were measured three times per participant, and the average was recorded to minimize measurement errors.

### Confounding factors control

To minimize potential confounding, the multiple regression models included variables related to demographic characteristics, socioeconomic status, and comorbidities. Demographic variables considered included age, sex, and household composition, while socioeconomic factors included level of education, marital status, and main occupation. Comorbidities, such as HIV status and other chronic illnesses, were also included as covariates.

### Data analysis

Data were analysed using SPSS Version 24. Dietary intake data were captured and analysed using the NutriSurvey computer program, with nutrient intake calculations based on the Kenya Food Composition Tables (Food and Agriculture Organization and Government of Kenya, [8]). Descriptive statistics were used to summarize variables. Chi-square tests assessed associations between categorical variables, while Pearson’s correlation assessed relationships between continuous variables. Variables significant at bivariate analysis (*p* < 0.05) were included in the multiple linear regression model to identify independent predictors of nutritional status. A p-value of less than 0.05 was considered statistically significant.

### Ethical considerations

Ethical approval was obtained from the Kenyatta University Ethics Review Committee (Approval No: PKU/2685/11809). Written informed consent was obtained from all participants before data collection. The consent form was available in English and Kiswahili, and verbal explanations were provided where necessary to accommodate varying literacy levels.

## Results

### Demographic and socio-demographic characteristics of TB patients

A total of 141 adult pulmonary TB patients participated in the study. The majority were male (59.6%), with a mean age of 33.9 ± 12.6 years. Most participants were aged 25–34 years (39.0%), followed by 18–24 years (22.0%). Regarding marital status, 48.2% were married, 27.0% had never married, 9.2% were living together, and 15.6% were separated or divorced. Household composition varied, with respondents living with children (29.3%), spouses (26.6%), alone (26.6%), other relatives (12.8%), or parents (5.3%). In terms of education, 34.0% had completed secondary education, 31.9% had completed college or tertiary education, 22.0% had completed primary education, and a minority had no formal education (7.1%) or pre-primary education (0.7%) (Table 1).

**Table 1:** Demographic and socio-economic characteristics of TB patients

Characteristics	Total (*n* = 141) *n*(%)
Sex (male)	84(59.6%)
Mean age	33.86 + 12.60
Age	
18–24	31(22.0%)
25–34	55(39.0%)
35–44	24(17.0%)
45–54	15(10.6%)
55–64	12(8.5%)
65 and above	4(2.8%)
Marital status	
Never married	38(27.0%)
Married	68(48.2%)
Living together	13(9.2%)
Separated/Divorced	22(15.6%)
Household composition	
Children	55(29.7%)
Parents	7(3.8%)
Relatives	23(12.4%)
Spouse	48(25.9%)
Alone	52(28.1%)
Level of education	
None - pre primary	10(7.1%)
Primary	31(22.0%)
Post-primary, vocational	1(0.7%)
Secondary education	48(34.0%)
College (middle level)	45(31.9%)
University level	6(4.3%)

### Dietary practices of TB patients

#### Meal frequency and pattern of TB patient

The mean meal frequency was 3.22 ± 1.28 meals/day, with approximately one-third (36.2%) reporting skipping at least one main meal. Reasons for skipping meals included lack of money (45.1%), lack of time (33.3%), not being hungry at mealtime (15.7%), and personal preference (5.9%) (Table 2). Breakfast, lunch, and supper were consumed by 77.3%, 63.8%, and 97.2% of participants, respectively (Table 2).

**Table 2:** Meal frequency and meal patterns of TB patients

Characteristics	Total
Meal frequency (meals/day)	3.22 + 1.28
Skipped main meals	51(62%)
Reasons for skipping:	
Lack of time	17(33.3%)
Lack of hunger	8(15.7%)
Lack of money	23(45.1%)
Preference	3(5.9%)
Meal patterns:	
Breakfast	109(77.3%)
Lunch	90(63.8%)
Supper	137(97.2%)

#### Food consumption pattern

**Fig. 1:**
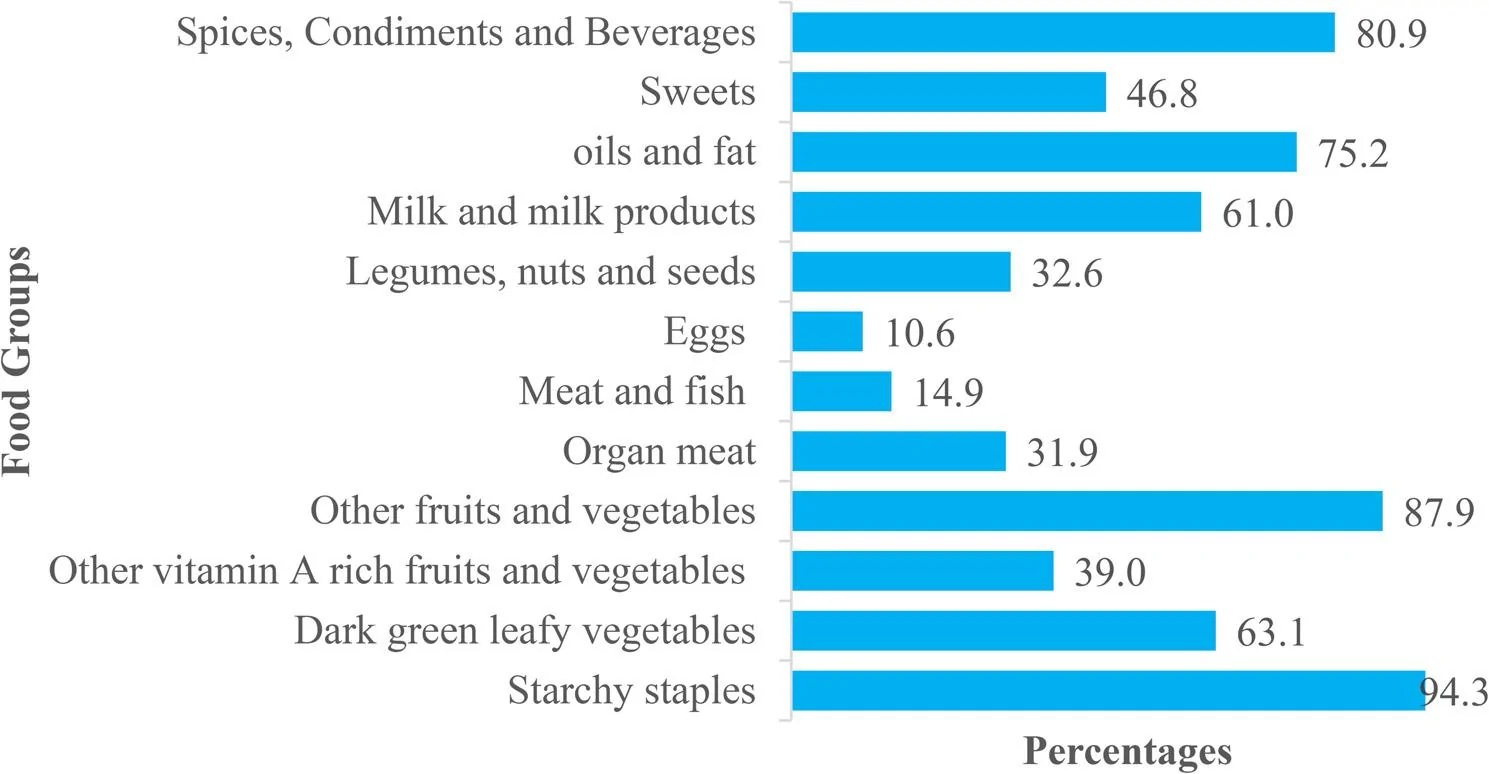
Food consumption pattern

The majority (94.3%) of respondents consumed starchy staples, making them the most commonly consumed food group. Other commonly consumed food groups included oils and fats (75.9%) and sweets (50.0%). Fruits and vegetables were also widely consumed, including other fruits and vegetables (87.9%), spices, condiments and beverages (80.9%), dark green vegetables (63.1%), and milk and milk products (61.0%). Protein-rich foods such as eggs (10.6%), meat and fish (14.9%), organ meats (31.9%), and legumes, nuts, and seeds (32.6%) were less frequently consumed (Fig. 1).

#### Nutrient adequacy of the TB patients

The mean energy intake for the study population was 2368 kcal/day for males and 2032 kcal/day for females. The energy intake of TB patients accounted for 90.0% and 88.3% of the RDA for TB for males and females, respectively. Carbohydrate contributed more to the daily caloric intake, with males consuming 91.2% of the RDA for TB patients and females taking 88.7% of the recommended dietary intake for TB patients. The study established that protein was least consumed macronutrient, even if it is one of the key nutrients that are required for the dietary management of TB. The mean protein intake was 61 ± 9.21 g/day, representing 64.2% of the daily intake for males and 48 ± 11.42 g/day, representing 58.5% for females. The mean fat intake was 62 g/day, which was 77.5% of the male RDA for TB and 56 g/day (80.0%) for the female RDA for TB. The study population did consume fibre, almost to the recommended dietary intake for TB patients; males averaged 93.3%, while females consumed about 96.7% of TB patients RDA (Table 3). Regarding micronutrients intake, the study reported that all the micronutrients were reported to have been consumed to below the recommended dietary intake (Table 3).

**Table 3:** Nutrient adequacy for male and female TB patients

Characteristic	*RDA for TB patients		Dietary intake (% of RDA)	
	Male	Female	Male (*n* = 84)	Female (*n* = 57)
Energy (kcal)	2630	2300	2368(90.0%)	2032(88.3%)
Carbohydrates g/day	396	336	361(91.2%)	298(88.7%)
Protein g/day	95	82	61(64.2%)	48(58.5%)
Fat (g/day)	80	70	62(77.5%)	56(80.0%)
Fiber (g/day)	30	30	28(93.3%)	29(96.7%)
Vit A(µg)	900	700	581(64.6%)	559(79.9%)
Vitamin D (µg)	15	15	10(66.7%)	11(73.3%)
Vit.E (mg)	13	13	9(69.2%)	10(76.9%)
Vit B1 (mg)	1.5	1.1	1.2(80.0%)	1(90.9%)
Vit B2 (mg)	1.6	2.4	1.1(68.8%)	1.9(79.2)
Vit. B 6 (mg)	1.6	1.3	1(62.5%)	1.1(84.6%)
Folate(µg)	500	400	368(73.6%)	344(86.0%)
Vit B3 (mg)	20	16	14(70.0%)	13(81.3%)
Vit. B12 (µg)	3	2.4	1.9(63.3%)	2.1(87.5%)
Vit C(mg)	90	75	58(64.4%)	59(78.7%)
Calcium (mg)	1250	1000	879(70.3%)	665(66.5%)
Magnesium (mg)	325	220	254(78.2%)	194(88.2%)
Phosphorus (mg)	1000	800	766(76.6%)	698(87.3%)
Iron (mg)	8	18	7(87.5%)	11(61.1%)
Selenium (ug)	55	55	39(70.9%)	39(70.9%)
Zinc(mg)	14	12	9(64.3%)	8(66.7%)

*RDA values are based on guidelines for management of tuberculosis and leprosy in Kenya, July 2013

### Nutritional status of adult TB patients

#### Nutritional status of TB patients by gender

The mean BMI was 19.52 ± 2.98 for males and 21.49 ± 4.66 for females, indicating a higher average BMI among females. Among males, 35.7% were underweight, 59.5% had normal BMI, and 4.8% were overweight. Among females, 35.1% were underweight, 50.9% had normal BMI, and 14.0% were overweight. No participants were classified as obese (Table 4).

**Table 4:** Nutritional status of TB patients by gender

Gender	Mean BMI ± SD	Underweight (%)	Normal (%)	Overweight (%)
Male (*n* = 84)	19.52 ± 2.98	35.7	59.5	4.8
Female (*n* = 57)	21.49 ± 4.66	35.1	50.9	14.0

#### Nutritional status by age category

The distribution of BMI across age categories showed that underweight prevalence decreased with increasing age, while overweight prevalence increased. Age was significantly associated with nutritional status (χ² = 26.885, df = 10, *p* = 0.030) (Table 5).

**Table 5:** Nutritional status by Age Category

Age Category	Underweight (%)	Normal (%)	Overweight (%)
18–24	45.2	54.8	0.0
25–34	30.9	61.8	7.3
35–44	37.5	54.2	8.3
45–54	20.0	53.3	26.7
55–64	16.7	50.0	33.3
≥ 65	25.0	75.0	0.0

**χ² = 26.885**,** df = 10**,*p* = 0.030

Statistical significance was considered at *p* < 0.05

#### Nutritional status in relationship to selected social demographic characteristics

Associations between the selected socio-demographic characteristics and BMI were analyzed using chi-square tests (Table 6). Sex (χ² = 10.429, *p* = 0.511) and marital status (χ² = 18.096, *p* = 0.106) were not significantly associated with nutritional status. However, living arrangements (χ² = 23.093, *p* = 0.003), level of education (χ² = 21.676, *p* = 0.017), and main occupation (χ² = 20.658, *p* = 0.024) were significantly associated with BMI among the study participants, suggesting that these factors may influence nutritional outcomes.

**Table 6:** Selected Socio-Demographic Characteristics in Relation to Nutritional status

Characteristic	χ²	df	*p*-value
Sex	10.429	2	0.511
Marital status	18.096	6	0.106
Household composition	23.093	6	0.003
Level of education	21.676	10	0.017
Main occupation	20.658	10	0.024

### Dietary practices in relation to nutritional status

A significant correlation was found between the frequency of meals consumed and nutritional status (BMI) for study participants (χ^2^ = 30.429, *p* = 0.001). A chi square analysis reported a significant relationship between the pattern of consumption of lunch with nutritional status (BMI) among the TB patients (χ2 = 22.238, df = 2, p-value = 0.001) (Table 7). Additionally, a significant relationship between consumption of starchy staples, other fruits and vegetables, organ meat, meat and fish, eggs food groups, and nutritional status (BMI) (p ˂ 0.05). The study did not report a significant relationship between the dark green leafy vegetables, other vitamin A rich fruits and vegetables, legumes, nuts and seeds, milk and milk products, oils and fat, sweets and spices, condiments, and beverages food groups with nutritional status BMI (p ˂0.05) (Table 7).

Pearson’s product-moment correlation coefficient found a significant association between the amount of energy (*r* = 0.421, p-value = 0.019), carbohydrate (*r* = 0.342, p-value = 0.029), and protein (*r* = 0.312, p-value = 0.042) consumed by respondents and their BMI. Pearson’s product-moment correlation coefficient found a significant relationship between the amount of iron (*r* = 0.233, p-value = 0.001) and selenium (*r* = 0.722, p-value = 0.001) consumed by respondents and their BMI.

**Table 7:** Food consumption pattern in relation to nutritional status

Characteristics	Χ2	Df	*p* value
Meal frequency	30.429	10	0.001
Skipped main meals	4.241	2	0.123
Meal pattern			
Regular breakfast day	2.775	2	0.051
Regular Lunch	22.238	2	0.001
Regular supper	3.046	2	0.123
Food consumption pattern			
Starchy staples	7.706	2	0.021
Dark green leafy vegetables	8.608	4	0.072
Other vitamin A rich fruits and vegetables	4.998	2	0.076
Other fruits and vegetables	25.856	2	0.001
Organ meat	8.829	2	0.012
Meat and fish	15.242	2	0.001
Eggs	17.087	1	0.001
Legumes, nuts and seeds	2.138	2	0.343
Milk and milk products	3.143	1	0.208
oils and fat	4.013	2	0.437
Sweets	1.574	2	0.445
Spices, Condiments and Beverages	43.909	2	0.501

NB. Vs represents versus

Statistical significance was considered at *p* < 0.05

### Determinants of nutritional status

Multiple linear regression was performed to identify independent predictors of nutritional status (BMI) among adult pulmonary TB patients. Variables included in the model were those that showed significant associations in bivariate analyses, namely age, household composition, meal frequency, total energy intake, and regular lunch consumption. Other factors, including level of education and consumption of certain food groups (starchy staples, other fruits and vegetables, organ meat, meat and fish, and eggs), were associated with BMI in univariate analyses but did not independently predict BMI in the adjusted model and were therefore not included as predictors.

The regression model was statistically significant, explaining approximately 42% of the variation in BMI (Adjusted R² = 0.42, F(6,134) = 16.3, *p* < 0.001). Older age, living alone, higher meal frequency, higher total energy intake, and regular lunch consumption were independently associated with higher BMI (Table 8). Specifically, participants aged 25–34, 35–44, 45–54, and 55–64 years had significantly higher BMI compared to those aged 18–24 years (β = 1.57–2.85, all *p* < 0.05). Household composition was also significant: participants who lived with others had lower BMI compared to those who lived alone (β = − 1.43, 95% CI: − 2.21 to − 0.65, *p* < 0.001). Dietary factors independently associated with BMI included meal frequency (β = 1.217, 95% CI: 0.773–1.661, *p* < 0.001), total energy intake (β = 0.003 per kcal, 95% CI: 0.002–0.005, *p* < 0.001), and regular lunch consumption (β = 0.94, 95% CI: 0.19–1.70, *p* = 0.015) (Table 8).

Although other factors, such as education level and consumption of specific food groups (starchy staples, other fruits and vegetables, organ meat, meat and fish, eggs), showed some associations in univariate analyses, they were not included in the regression model, as they did not independently predict BMI after adjusting for other variables.

**Table 8:** Multiple linear regression analysis of determinants of nutritional status (BMI) among adult pulmonary TB patients (*N* = 141)

Predictor	β (Coefficient)	95% CI	*p*-value
Age 25–34 vs. 18–24 (ref)	1.57	0.38–2.76	0.01
Age 35–44 vs. 18–24 (ref)	2.32	0.69–3.95	0.005
Age 45–54 vs. 18–24 (ref)	2.85	1.12–4.57	0.002
Age 55–64 vs. 18–24 (ref)	1.94	0.21–3.66	0.028
Age ≥ 65 vs. 18–24 (ref)	2.12	–0.46–4.70	0.105
Living with others vs. living alone (ref)	–1.43	–2.21 – – 0.65	< 0.001
Meal frequency (meals/day)	1.217	0.773–1.661	< 0.001
Total energy intake (kcal/day)	0.003	0.002–0.005	< 0.001
Regular lunch (yes vs. no)	0.94	0.19–1.70	0.015

Statistical significance was considered at *p* < 0.05

## Discussion

### Demographic and socio-economic characteristics of TB patients

#### Sex of the study participants

In this study, approximately 59.5% of the participants were male, which is consistent with global findings. For instance, the WHO Global Report (2023) and studies in Pokhara, India [9] and Turkana County, Kenya(Nthiga, Mbithe, Mugendi, Nyangaresi, et al., [10]) reported similar trends, with a higher prevalence of tuberculosis among males. The higher TB prevalence in males could be related to biological factors, higher exposure rates, and sociocultural practices [1].

#### Age

The age distribution of this study’s participants aligns with findings from previous studies, including one in Pokhara, India, where the majority were between 20 and 30 years of age [9], and reports from Kenyan demographic surveillance that highlight a high TB burden in people aged 20–44 (Centre for Health Solutions – Kenya, [11]). TB burden among those aged 20–44. This reflects the high prevalence of TB in young adults, who are more socially active, leading to increased exposure. However, the findings of lower TB prevalence in the 65 + age group diverged from the Ministry of Health’s 2017 survey, which noted a higher prevalence among the elderly. This could suggest evolving risk factors or improvements in the management of TB in older populations.

#### Marital status

Most participants were married, aligning with studies in Ethiopia indicating a high proportion of TB patients being married [12]. Similar trends were observed in China, where married adults accounted for a significant proportion of TB cases [13], suggesting marital status may be intertwined with household exposure risk and care-seeking behaviours.

#### Household composition

The majority of respondents reported living with children, spouses, or other family members, with about one-quarter living alone. Interestingly, TB patients who lived alone had better nutritional status than those living with multiple household members. This finding contradicts the meta-analysis by Ho et al., [14], which suggested that larger family sizes generally provide more social support, better access to nutritious food, and higher adherence to health-promoting behaviours.

The discrepancy in our study could be explained by resource dilution in larger households, where limited food and economic resources must be shared among many members, potentially reducing the quantity and quality of meals available per person [15, 16]. Moreover, households experiencing food insecurity may reduce meal frequency or portion sizes as a coping strategy, leading to skipped meals or irregular consumption patterns, which negatively affect nutritional status [17]. In contrast, individuals living alone may have greater control over their dietary choices and meal frequency, enabling them to maintain better nutrition despite social isolation.

These differences highlight that the effects of household composition on nutrition are context-specific and may depend on economic conditions and food access. Overall, these findings underscore the importance of considering household food security when interpreting meal frequency patterns and their impact on nutritional status among TB patients. TB patients in food-secure households may have consistent access to adequate meals, while those in food-insecure households are more likely to skip meals, contributing to undernutrition.

#### Level of education

Most respondents had primary or secondary education, consistent with the 2019 Kenya Population and Housing Census (Kenya National Bureau of Statistics, [18]). However, this pattern differs from findings in Vihiga County, Kenya, where lower educational attainment was reported among tuberculosis patients [19]. The findings are also comparable to studies from Indonesia, where primary education predominated [20]. This variation across settings may reflect underlying socio-economic and contextual differences. Importantly, education is closely linked to health literacy, which influences patients’ ability to understand nutritional recommendations, adhere to treatment, and make informed dietary choices. Therefore, the observed education profile in this study may have implications for the design of targeted nutrition education and TB management interventions.

### Dietary practices of TB patients in Kiambu County

#### Meal frequency and meal patterns

There was a high prevalence of meal skipping, particularly lunch, driven by financial constraints, time scarcity, and personal preferences — consistent with studies in Kiambu and Turkana Counties (Wambui Mary Kamwana et al., [21];Nthiga et al, [7]). Meal frequency was significantly associated with nutritional status, reinforcing the role of food security: households with unstable food access are more likely to skip meals, which directly impacts BMI and overall nutritional health [17].

#### Distribution of food groups consumed in 24-hour recall

The study found that respondents’ diets were heavily reliant on starchy foods, fruits, and milk, while protein intake was limited an important concern given the role of protein in supporting immune function and TB management. These findings are consistent with observations in Kiambu, Kenya (Wambui Mary Kamwana et al., [21])and align with global evidence indicating that protein-rich foods are often less accessible due to economic constraints. The low consumption of protein-rich foods may compromise recovery, reduce treatment efficacy, and increase susceptibility to opportunistic infections among TB patients. This dietary pattern highlights the need for targeted nutrition interventions, including education on affordable and culturally acceptable protein sources, as well as broader strategies to improve access to protein-rich foods in resource-limited settings.

#### Dietary intake of the study participants

The study reported consumption of both energy, macronutrients and most of micronutrients to below the recommended RDA. The study findings align with research conducted in China regarding nutritional intakes and associated factors among tuberculosis patients, which indicated that 87.4% of male patients and 59.9% of female patients did not meet adequate energy consumption levels. A majority of male (90.8%) and female (58.4%) tuberculosis patients exhibited inadequate daily protein consumption. Further analyses indicated that the mean daily intakes of several micronutrients were inadequate for the majority of patients [13]. The study aligns with research conducted in China regarding the association between dietary micronutrient intake and the treatment failure rate of pulmonary tuberculosis. It reported low consumption of vitamin C and zinc, both of which were linked to symptoms of TB disease [22]. Similarly, a study conducted in Turkana County, Kenya, revealed inadequate intake of energy and macronutrients, as well as insufficient consumption of essential micronutrients for tuberculosis management. Vitamins A, D, B1, B2, B6, C, zinc, selenium, and folate were consumed at levels below the recommended dietary allowance despite their important role in TB treatment [7].

#### Nutritional status of the TB patients

Most participants had normal nutritional status, in contrast to findings from Turkana County where undernutrition was predominant [7]. This disparity could be attributed to regional differences in food availability, socioeconomic conditions, and access to healthcare.

#### Relationship between social demographic characteristics and nutritional status

Age was significantly associated with BMI, indicating improved nutritional status with increasing age, possibly due to accumulated dietary habits and more stable income sources. Younger individuals may experience higher energy expenditure and irregular meal patterns due to work and lifestyle demands, which can affect BMI. Previous studies have also shown that socio-demographic factors such as age influence dietary behaviour and nutrition-related outcomes [23].

The relationship between household composition and poor nutrition underscores the influence of food distribution dynamics and resource competition in larger households. Evidence suggests that household food insecurity and crowding can negatively influence individual dietary intake and nutritional outcomes because food resources must be shared among more members, reducing per-capita consumption [24]. Education level was significantly related to BMI, supporting studies showing that higher education is associated with improved nutrition knowledge and healthier dietary choices [25]. Education enhances individuals’ ability to access, understand, and apply health information, which can influence dietary behaviour and nutritional outcomes. Furthermore, nutrition and health education interventions have been shown to improve dietary practices and nutritional status among patients, including those with tuberculosis [26].

#### Relationship between selected dietary practices and nutritional status

A significant relationship was observed between meal frequency and nutritional status, consistent with findings from Lodwar, Turkana County [7], and Pokhara, Nepal [9]. Patients with higher meal frequency had better BMI, reinforcing the importance of consistent and adequate dietary intake in TB management.

The observation that regular breakfast and lunch consumption was associated with better BMI aligns with research conducted in Kiambu County, where (Wambui Mary Kamwana et al., [10]) reported that individuals who consumed regular meals were less likely to experience malnutrition and had better overall nutritional status. In contrast, Aishwarya and Shekhar [27] observed in their study in Mumbai, India, that skipping meals was paradoxically associated with higher BMI, which they attributed to possible compensatory overeating, irregular dietary patterns, and cultural differences in eating habits. These contrasting findings suggest that the relationship between meal frequency and nutritional status may vary across settings depending on lifestyle, dietary practices, and socio-economic conditions.

#### Determinants of nutritional status

The regression analysis revealed that older participants were more likely to have a normal nutritional status suggesting that life stage and possibly accumulated dietary habits influence nutrition. This observation aligns with a study conducted in Ethiopia, which found that TB patients aged 18 to 24 years had a fourfold higher risk of undernutrition compared to those aged 45 years and above [28]. Additionally, meal frequency and calorie intake emerged as key determinants, reinforcing the importance of consistent and adequate dietary intake. Research from Ethiopia supports this, indicating that low dietary diversity significantly increases the odds of undernutrition among adult TB patients [7]. Living arrangements also appeared to impact nutrition, indicating that social support and household dynamics may influence dietary behaviours. A study in Ghana found significant associations between nutritional status and factors such as age, education level, and employment status, suggesting that socio-demographic elements play a crucial role in the nutritional well-being of TB patients [9].

## Conclusion

This study demonstrates that dietary practices, particularly regular meal consumption and adequate caloric intake, are important determinants of the nutritional status of adult pulmonary tuberculosis (TB) patients. Socio-demographic factors such as age and household composition were also associated with nutritional outcomes, indicating that household dynamics and economic conditions may influence food access and dietary intake.

These findings highlight the need to integrate nutrition-focused interventions, including dietary counseling, meal planning, and targeted supplementation, into TB care programs. Strengthening nutrition support within TB management may help improve patient recovery and treatment outcomes. Future research should further examine effective strategies to address barriers to adequate food consumption among TB patients in similar settings.

### Recommendation

To improve the nutritional status of TB patients, interventions should focus on promoting regular meal consumption, structured meal planning, and improved food accessibility, particularly among patients from resource-constrained households. Strengthening community-based nutrition education and family support systems may further encourage healthy dietary practices and improve dietary intake during treatment. In addition, integrating routine nutritional assessment, counseling, and targeted supplementation into TB treatment programs is essential to ensure comprehensive patient care and improve treatment outcomes.

## Supplementary Information

Supplementary Material 1.

Supplementary Material 2.

## Data Availability

The data generated and/or analysed, as well as the materials used, including the questionnaires, during the current study, are available from the author upon reasonable request.
