# Supplementary material for: Dietary practices and nutritional status of adult pulmonary tuberculosis patients in Kiambu level five hospital, Kiambu county, Kenya
Source: BMC Nutr. 2026 Jul 8;12:172. doi: 10.1186/s40795-026-01346-9 (PMC13491660; doi:10.1186/s40795-026-01346-9)
Supplement: Supplementary file 1 [file 40795_2026_1346_MOESM1_ESM.pdf]

APPENDIX 1: RESEARCHER ADMINISTERED QUESTIONNAIRE

Interviewer's note: There are six sections in this baseline questionnaire.
Each component should be completed.

Nutrition and health information of tuberculosis patients 18 years and above at Kiambu County
Part A: Interview record
Part B: Demographic and Social Economic Information
Part C: Dietary practices
Part D: Nutrition assessment

PART A: INTERVIEW RECORD

Questionnaire number …………… The interviewer's name …………………………
Residence of the despondence ……………………Interview date (dd/mm/yyyy): __ __/__ __/ _
Signed informed consent obtained? 1 = Yes (continue the interview) 2 = No (terminate the interview)
Month after TB diagnoses [ ] Time started ………………Time ended…..…………………

**PART B: SOCIAL DEMOGRAPHIC AND ECONOMIC INFORMATION**		
B1.	Date of birth<br>***Write the date of birth in the space provided.***<br>***Confirm age from ID***	__ ___/__ ___/___ ___
B2.	Sex of the respondent	1. Male 2. Female
B3.	Marital status?<br><br>***Tick/circle only one option***	1.Never married<br>2.Married<br>3.Living together<br>4.Separated/Divorced<br>5.Windowed
B4.	What was the highest level of education you ever completed?<br><br>***Tick/circle only one option***	a) Primary<br>b) Post-primary, vocational<br>c) Secondary, a level<br>d) College (middle level)<br>e) University<br>f) Others (specify)………
B5.	Whom do you live with?<br>***Tick/circle only one option***	1)Children 2) Parents 3) Relatives<br>4) Spouse 5) Well-wishers
B6.	How many people live with you in the house?	Number ___ ___
B7.	What is your main occupation?	1. Casual labour<br>2. Farmer<br>3. Labour (salaried)<br>4. Business<br>5. Unemployed<br>6. Housewife

	Tick/circle only one option	7. Domestic help<br>8. Others (specify)………
B8.	What is the main source of family food?	1. Purchase<br>2. Farm Production<br>3. Borrowing<br>4. Food Aid/Donations<br>5. Others (specify)………
B9.	What is your average monthly income?	Ksh. _______________
B10.	Do you own;<br>***Tick/circle as many options as responded***	Yes No<br>1) A bicycle 1 2<br>2) A motorcycle or motor scooter 1 2<br>3) An animal-drawn cart 1 2<br>4) A car or truck 1 2<br>5) A boat with a motor 1 2
B11.	Do you own any agricultural land?<br>***(if Yes go to B8, If No, go to B9)***	Yes . . . . 1<br>No. 2
B12.	How many hectares of land (altogether) do you own?<br>***Write No. hectare in figures)***	No of hectares _______
B13.	Do you live in a:<br>***Tick/circle only one option***<br><br>***If RENTED****, go to question B7* ***If NOT*** *rented, skip to question B8.*	1.Rented house<br>2.Own house in parental compound<br>3.Own house in own compound
B14.	If rented, how much rent do you pay per month? ***(Write the amount in figures)***	Ksh.______
B15.	How many of the following animals do you own?<br>***"if none, write '00'. if more than 95, write '95'if unknown, write '98'."***	1. Cattle (indigenous)..........[ ][ ]<br>2. Cows/bulls……………...[ ][ ]<br>3. Horses/donkeys/mules.…[ ][ ]<br>4. Goats…………………....[ ][ ]<br>5. Sheep…………………...[ ][ ]<br>6. Chicken…………………[ ][ ]
B16.	Which fuel source does your home utilize **most** frequently for cooking?<br><br><br>***(Tick/circle one option)***	1. Kerosene<br>2. Electricity<br>3. Firewood<br>4. Natural gas and/or LPG<br>5. Charcoal<br>6. Agricultural crop<br>7. Other (specify)
B17.	What is your **main** source of light?<br><br><br>***Tick/circle one option***	1. Kerosene<br>2. Electricity<br>3. Solar<br>4. Candle<br>Others (Specify)…………….
B18.	What is the number of rooms in your	No. of rooms __________

	house? ***(Write No. of rooms in figures)***	
B19.	How many bedrooms are utilized for sleeping in the household?<br>***(Write No. of bedrooms in figures)***	No. of bedrooms ___________
B20.	What **key** component makes up the flooring in your houses?<br>***Tick/circle one option***	1) Cement 2) Sand 3) Carpet 4) Dung<br>5) Ceramic tiles 6) Wood planks<br>7) Others ....
B21.	What is the main material of the roof?<br>***Tick/circle one option***	1. Thatch/*Makuti* 2. Dung/Mud 3. Corrugated iron *(Mabati)* 4. Tin cans 5. Asbestos sheet 6.Concrete Tiles 7.Others (specify)..............
B22.	What is the main material of the wall?<br>***Tick/circle one option***	1). Cane/palm/trunks 2). Dirt 3). No wall 4). Bamboo with mud 5). Stone with mud 5). Plywood 6). Cardboard 7). Reused wood 8). Cement 9). Stone with lime/cement 10). Bricks 11). Cement blocks 12). Wood planks/shingles

PART C: DIETARY PRACTICES

Now (Name) I am going to ask you questions about your dietary practices.

Section 1: Selected Dietary Practices

1. How would you describe your appetite?.....................
    a) Good
    b) Neutral
    c) Not good etc.
2. Do you eat at regular times each day
    a) Breakfast 1). Yes 2). No
    b) Lunch 1). Yes 2). No
    c) Supper 1). Yes 2). No

   (***Ask for each***)
3. How many meals do you typically eat each day …………..? what of weekends days……..?
4. How do you make your food richer with energy giving foods?
    1) Adding sugar/honey to porridge, tea, juice etc.
    2) Adding oil to foods
    3) Use of Ready to Use Therapeutic foods
    4) Use of Ready to Use Supplementary Foods

Section 2: A 24 Hour Recall

Now (Name) I am going to ask you questions about the foods eaten and drinks drank yesterday from the time you woke up to the time you went to sleep last evening.

A 24-hour recall form will be used to document the participant's food and beverage intake the day prior, whether it was at home or away from it, from the time they got up to the time they went to bed. The name of food/dish, the ingredients used, amount consumed in household measure and in metric measure (gram) will be will be recorded. Verify whether the day was usual or unusual.
1= usual day 2= unusual
If unusual, describe why;
1.=Celebration 2=Religious activity 3=Little food in household 4=Other (specify)

Meal/Time	Name of dish/food	Description of ingredients	Amt. consumed (HH measures)	Quantity in grams	Method of preparation
Breakfast					
Mid-morning					
Lunch					
Afternoon snack					
Evening snack					
Supper					

NB. Method of preparations include a) Boiling b) stewed c) Roasting d) Deep frying e) Shallow frying f) Others (specify)

Section 3: Individual Dietary Diversity Baseline Questionnaire for Women respondents

Based on the 24-hour recall data listed above, complete the food groups. A score of '1' to be assigned if a food from that group was consumed at least once, regardless of the quantity. A score of '0' to be assigned to any food group that was not consumed.

Ask the respondent whether they ate any food items from any food groups that were not mentioned (probe).

SNO.	Food group	Examples	**Score:** Yes = 1 No = 0
1.	**Starchy staples**	**Cereals:** Wheat, sorghum, millet, rice, corn/maize, or any other grains or products manufactured from these<br>**White root & Tubers;** White yams, white cassava, white potatoes, or other root-based meals	
2.	**Dark green leafy vegetables**	Vegetables with dark green leaves, including wild variety + *Vitamin A-rich leaves that are locally available like amaranth, cassava leaves, kale, spinach*	
3.	**Other vitamin A rich fruits and vegetables**	**Vegetables rich in vitamin A and tubers:** sweet potato, squash, pumpkin, or carrot which have orange interiors *& other readily accessible veggies high in vitamin A that are local*<br>**Vitamin a rich fruit**: ripe papaya, dried peach, fresh or dried apricots, ripe mango, cantaloupe, and other readily available vitamin A-rich fruits in these and other local juices.	
4.	**Other fruits and vegetables**	**Other Vegetables**: Other veggies (such as tomato, onion, and eggplant) and additional vegetables that are available nearby<br>**Other fruits:** Other fruit varieties, such as wild fruit and 100% pure fruit juice made from them	
5.	Organ meat	meats from the liver, kidney, heart, or other organs, or blood-based foods	
6.	Meat and fish	Meats such as chicken, duck, hog, lamb, goat, rabbit, game, and insects. Shellfish and fish might be fresh or dried	
7.	Eggs	Any type of egg, including those from guinea hens, ducks, or chickens.	
8.	Legumes, nuts and seeds	legumes, peas, lentils, nuts, seeds, or products produced from them that are dried (e.g. hummus, peanut butter)	
9.	Milk & milk prdt	Yogurt, cheese, and other dairy items	

TOTAL SCORE (ADD SCORES FOR GROUP 1 – 9) -------------------

THE DIETARY DIVERSITY IS

1. Low dietary diversity (0 – 3)
2. Moderate dietary diversity (4 – 5)
3. High dietary diversity (6 -9)

(Circle the appropriate one based on the total scores and options 1 to 3)

Section 4: Household Dietary Diversity Questionnaire for adults TB respondents

Based on the 24-hour recall data listed above, complete the food groups. A score of '1' to be assigned if a food from that group was consumed at least once, regardless of the quantity. A score of '0' to be assigned to any food group that was not consumed.

Ask the respondent whether they ate any food items from any food groups that weren't mentioned (probe).

SNO.	Food group	Examples	**<u>Score:</u>** Yes = 1 No = 0
1.	**Starchy staples**	**Cereals:** Wheat, sorghum, millet, rice, corn/maize, or any other grains or products manufactured from these<br>**White root & Tubers;** White yams, white cassava, white potatoes, or other root-based meals	
2.	**Dark green leafy vegetables**	Vegetables with dark green leaves, including wild variety *+ Vitamin A-rich leaves that are locally available like amaranth, cassava leaves, kale, spinach*	
3.	**Other vitamin A rich fruits and vegetables**	**Vegetables rich in vitamin A and tubers:** sweet potato, squash, pumpkin, or carrot which have orange interiors *& other readily accessible veggies high in vitamin A that are local*<br>**Vitamin a rich fruit**: ripe papaya, dried peach, fresh or dried apricots, ripe mango, cantaloupe, and other readily available vitamin A-rich fruits in these and other local juices.	
4.	**Other fruits and vegetables**	**Other Vegetables**: Other veggies (such as tomato, onion, and eggplant) and additional vegetables that are available nearby<br>**Other fruits:** Other fruit varieties, such as wild fruit and 100% pure fruit juice made from them	
5.	Organ meat	meats from the liver, kidney, heart, or other organs, or blood-based foods	
6.	Meat and fish	Meats such as chicken, duck, hog, lamb, goat, rabbit, game, and insects. Shellfish and fish might be fresh or dried	
7.	Eggs	Any type of egg, including those from guinea hens, ducks, or chickens.	
8.	Legumes, nuts and seeds	legumes, peas, lentils, nuts, seeds, or products produced from them that are dried (e.g. hummus, peanut butter)	
9.	Milk and milk products	Yogurt, cheese, and other dairy items	
10.	Oils and fats	Butter, oil, or other fats used to cook with or add to meals	
11.	Sweets	Sugar, honey, sweetened beverages like soda or juice, as well as sweet meals like chocolate, sweets, pastries, and	

		cakes	
12.	Spices, condiments, beverages	condiments (soy sauce, hot sauce), spices (black pepper, salt), coffee, tea, and alcoholic beverages	

TOTAL SCORE (ADD SCORES FOR GROUP 1 – 12) -------------------

PART D: NUTRITION ASSESSMENT
Anthropometric measurements information

**Measurement**	**1st**	**2nd**	**Average**	**BMI (KG/H^2)**	**Nutrition Status** (Indicate codes as in the Key)
Weight (to the nearest 0.1 Kg)					
Height (to the nearest 0.1 cm)					

Nutrition status key for BMI:
1. Severe thinness <16.00
2. Moderate thinness 16.00 - 16.99
3. Mild thinness 17.00 - 18.49
4. Normal range 18.50 - 24.99
5. Overweight ≥25.00
