# Supplementary material for: Dietary practices and nutritional status of adult pulmonary tuberculosis patients in Kiambu level five hospital, Kiambu county, Kenya
Source: BMC Nutr. 2026 Jul 8;12:172. doi: 10.1186/s40795-026-01346-9 (PMC13491660; doi:10.1186/s40795-026-01346-9)
Supplement: Supplementary file 2 [file 40795_2026_1346_MOESM2_ESM.pdf]

s/no	Item requested	Corrections made
1.	Questionnaire	The author developed the questionnaire.<br>It’s now uploaded and cited in the manuscript as appendix 1
2.	Consent for publication	Not Applicable”
